# Urine Glycoprotein Profile Reveals Novel Markers for Chronic Kidney Disease

**DOI:** 10.1155/2011/214715

**Published:** 2011-10-10

**Authors:** Anuradha Vivekanandan-Giri, Jessica L. Slocum, Carolyn L. Buller, Venkatesha Basrur, Wenjun Ju, Rodica Pop-Busui, David M. Lubman, Matthias Kretzler, Subramaniam Pennathur

**Affiliations:** ^1^Division of Nephrology, University of Michigan, Ann Arbor, MI 48105, USA; ^2^Department of Pathology, University of Michigan, Ann Arbor, MI 48105, USA; ^3^Division of Metabolism, Endocrinology and Diabetes, Department of Internal Medicine, University of Michigan, Ann Arbor, MI 48105, USA; ^4^Department of Computational Medicine and Biology, University of Michigan, Ann Arbor, MI 48105, USA; ^5^Department of Surgery, University of Michigan, Ann Arbor, MI 48105, USA

## Abstract

Chronic kidney disease (CKD) is a significant public health problem, and progression to end-stage renal disease leads to dramatic increases in morbidity and mortality. The mechanisms underlying progression of disease are poorly defined, and current noninvasive markers incompletely correlate with disease progression. Therefore, there is a great need for discovering novel markers for CKD. We utilized a glycoproteomic profiling approach to test the hypothesis that the urinary glycoproteome profile from subjects with CKD would be distinct from healthy controls. N-linked glycoproteins were isolated and enriched from the urine of healthy controls and subjects with CKD. This strategy identified several differentially expressed proteins in CKD, including a diverse array of proteins with endopeptidase inhibitor activity, protein binding functions, and acute-phase/immune-stress response activity supporting the proposal that inflammation may play a central role in CKD. Additionally, several of these proteins have been previously linked to kidney disease implicating a mechanistic role in disease pathogenesis. Collectively, our observations suggest that the human urinary glycoproteome may serve as a discovery source for novel mechanism-based biomarkers of CKD.

## 1. Introduction

Chronic kidney disease (CKD) affects approximately 11% of the US population with over 100,000 individuals progressing to end-stage renal disease (ESRD) annually [1, 2]. Despite this significant and growing public health problem, it remains difficult to predict which individuals will progress to ESRD. As ESRD carries a substantial increase in morbidity and mortality, it is critical to identify this high-risk patient population that would most benefit from early and aggressive therapy.

Current strategies for predicting CKD progression are limited. Pathologic examination of renal tissue provides valuable information on degree of interstitial fibrosis and predilection for ESRD. However, renal biopsy is invasive with a limited role for longitudinal followup. Quantitative measures of proteinuria have long been used as noninvasive markers of CKD progression [3], yet these largely albumin-based methods detect nonselective proteinuria and incompletely correlate with disease. With recent advances in high through-put technology and mass spectrometry techniques, urine proteomic investigation is an attractive tool in the pursuit for noninvasive and specific markers of CKD progression [4, 5].

Numerous investigators have successfully applied broad-scale urine proteomic strategies to kidney disease. The urine proteome predicts nephropathy and decline in renal function in diabetic subjects [6, 7]. It also correlates with early changes of focal segmental nephrosclerosis [8], can identify IgA nephropathy and renal allograft rejection [9, 10], and predicts treatment response and disease activity in nephrotic syndrome and lupus nephritis [11, 12]. Despite these advances, analysis of the entire urine proteome is particularly difficult in CKD. With disruption of the glomerular filtration barrier and leakage of abundant plasma proteins into the urine, a nonselective, largely albumin predominant, pattern often results [13]. To overcome this, methods to increase the detection of low-abundance proteins have been developed to provide disease specificity and clinical relevance of urine profiling and to mechanistically understand factors influencing disease progression. 

Glycoprotein enrichment techniques allow depletion of albumin and other abundant plasma proteins while providing a more thorough analysis of a subfraction of the urine proteome. As glycosylated proteins are critical for cellular interactions and signaling cascades, disease states are likely to cause early and specific alterations in urinary glycoprotein excretion. Indeed, glycoproteins are now important markers of autoimmunity and malignancy [14, 15]. More recently, the plasma glycoproteome has been used to predict nephropathy in diabetic subjects [16]. Despite this promising role as a noninvasive and specific biomarker of disease, little is known about the urinary glycoproteome in CKD. 

We hypothesized that the urinary glycoproteome would be altered in CKD compared to healthy controls and that specific glycoprotein alterations might be useful in predicting CKD progression. The overall goal of this study was to perform an initial exploratory analysis of the urine glycoproteins in patients with CKD compared to healthy controls. We present a comprehensive profiling of the urinary glycoproteome in control and CKD subjects utilizing a hydrazide enrichment technique combined with tandem mass spectrometry identification of the glycoproteins. 

## 2. Methods

### 2.1. Sample Collection and Processing

Clean catch urine samples were obtained from six CKD subjects and six age-matched healthy controls following written informed consent approved by the University of Michigan Institutional Review Board. Samples were stored at −80°C and thawed immediately prior to proteomic analysis. An initial 5000 g centrifugation was performed at 4°C for 10 minutes to remove cellular debris. Approximately, 30–50 mL healthy control samples and 1-2 mL CKD samples were concentrated using a 3 kDa filter cut-off membrane (Vivaspin 3 kDa MWCO, GE healthcare, Buckinghamshire, UK and Amicon ultra 0.5 mL, Millipore, Ireland resp.). As CKD subjects had higher urinary protein content (Table 1), the processed volumes were lower.

Urine protein concentration was determined using Coomassie Protein Assay Reagent with BSA standard (Thermo Scientific, Rockford, Illinois). 200 *μ*g of concentrated protein were utilized for downstream processing. Protein samples were exchanged into 50 mM ammonium bicarbonate buffer (pH 7.4). Urine creatinine concentration was determined by tandem mass spectrometry (MS/MS) as described previously by our group [17]. To determine the level of creatinine, a known amount of [^2^H_3_]creatinine was spiked into each sample. A full-scan mass spectrum revealed molecular ions of *m/z* 114 and 117 for authentic creatinine and [^2^H_3_]creatinine, respectively. The transitions of the *m/z* 114 to 44 and *m/z* 117 to 47 were monitored in multiple-reaction monitoring mode for authentic and [^2^H_3_]creatinine, respectively, utilizing an Agilent Technologies (New Castle, DE) 6410 Triple Quadrupole mass spectrometer system, equipped with an Agilent 1200 series HPLC system. The creatinine concentration in the urine sample was determined by comparing the peak areas for authentic and [^2^H_3_]creatinine for the above transitions.

### 2.2. Glycoprotein Separation and Enrichment

In order to assess recovery following the enrichment procedure, 5 *μ*g of invertase from *Saccharomyces cerevisiae* (Sigma, St. Louis, MO) was spiked into 200 *μ*g of protein in every sample. Glycoproteins were enriched from urinary proteins utilizing the hydrazide resin capture protocol as described previously by Zhang et al. [18]. Briefly, samples were oxidized with 10 mM sodium metaperiodate then incubated with hydrazide resin overnight at room temperature. Samples were then centrifuged at 3000 g for 2 minutes and the resin was washed successively with equal volumes 50 mM ammonium bicarbonate buffer (pH 7.4; Buffer A) supplemented with 8 M urea, followed by Buffer A alone and then water. The beads were resuspended in water, and the protein was reduced with 5 mm DTT followed by alkylation with 15 mM iodoacetamide. Trypsin (sequencing grade modified trypsin, Promega Corporation, Madison, WI) at 1 : 20 *μ*g ratio was added to the samples and incubated overnight at 37°C for digestion. Following digestion, the beads were centrifuged at 3000 g for 2 minutes and the resin was then washed successively with 1.5 M NaCl, 80% acetonitrile, 100% methanol, and Buffer A. The resin was then resuspended in Buffer A and incubated with 5 units of PNGaseF (New England Biolabs, Ipswich, MA) overnight at 37°C for glycopeptide release. The glycopeptides were cleaned using a reverse phase column and eluted with 50% acetonitrile/0.1% TFA followed by elution with 80% acetonitrile/0.1% TFA. The peptides were then dried at 60°C in a vacuum centrifuge and stored for mass spectrometric analysis.

### 2.3. Liquid Chromatography Electrospray Ionization (ESI/LC) MS/MS Analysis

Peptide samples were resuspended in 0.1% formic acid and loaded onto an in-house packed reverse phase separation column (0.075 × 100 mm, MAGIC C18 AQ particles, 5 *μ*m, Michrom Bioresources). The peptides were separated on a 1% acetic acid/acetonitrile gradient system (5–50% acetonitrile for 75 min, followed by a 10 min 95% acetonitrile wash) at a flow rate of ~300 nl/min. Peptides were directly sprayed onto the MS using a nanospray source. An LTQ Orbitrap XL (Thermo Fisher Scientific, Waltham, MA) was run in automatic mode collecting a high resolution MS scan (FWHM 30,000) followed by data-dependent acquisition of MS/MS scans on the 9 most intense ions (relative collision energy ~35%). Dynamic exclusion was set to collect 2 MS/MS scans on each ion and exclude it for an additional 2 min. Charge state screening was enabled to exclude +1 and undetermined charge states. 

### 2.4. Data Processing and Statistical Analysis

The Human UniProt database (Release 2011-5) was appended with a reverse database, a common contaminant list, and yeast invertase. Raw files were converted to mzXML format and searched against the database using X!Tandem with a *k-score* plug-in, an open-source search engine developed by the Global Proteome Machine (http://www.thegpm.org/). The search parameters were as follows: (1) precursor mass tolerance window of 100 ppm and fragment mass tolerance of 0.8 Da; (2) allowing two missed cleavages; (3) variable modification: oxidation of methionine (+15.9949 Da), carbamidomethyl cysteine (57.0214 Da), and +0.9840 Da, reflecting the conversion of asparagine in the NxS/T motif to aspartate due to the release of the N-linked glycopeptides from their oligosaccharides. All proteins with a ProteinProphet probability of greater than 0.9 were considered as positive identifications [19]. Only proteins containing peptides with the NxS/T sequence motif were included for statistical analysis.

Baseline characteristics of the control and CKD subjects were compared using Fisher's exact test for categorical variables and Student's *t*-test for continuous variables. Data is presented as means (±SD). Spectral counts for individual proteins were normalized to *Saccharomyces cerevisiae* invertase and to urine creatinine content. Spectral counts were compared across the two subject groups using the nonparametric Mann-Whitney test, and *P* values were adjusted for multiple comparisons using the False Discovery Rate (FDR) with reported *q*-values. All statistical analyses were performed with the use of SAS software, version 9.2. 

### 2.5. Gene Ontology Analysis

Significant proteins of interest were analyzed using the Gene Ontology Database (Gene Ontology Consortium, http://www.geneontology.org, Princeton University, New Jersey, US; [20]). For a given Gene Ontology (GO) category, the relative enrichment of genes encoding the proteins detected in CKD relative to all reference genes in that category were calculated as previously described using GO Tools made available by the Bioinformatics Group at the Lewis-Sigler Institute (Princeton University, New Jersey, US; [21]). A cutoff value of *P* < 0.01 was used to report a functional category as significantly overrepresented. To address the multiple comparisons problem that arises when many processes are evaluated simultaneously, the analysis included calculation of the FDR [21]. To improve statistical confidence in our results, all enriched functional categories were required to be significant using both methods (*P* < 0.01 and FDR < 0.05).

## 3. Results

### 3.1. Study Subject Characteristics

Urine was isolated from six subjects with CKD and six age-matched healthy controls. Baseline subject characteristics are provided in Table 1. Two important issues were considered with patient selection. First, the etiology of CKD was chosen to be diverse. This would ensure robustness of the putative markers as a CKD marker rather than a disease-specific marker. Second, we specifically targeted early Stage 3 CKD subjects to identify early disease markers that would potentially indicate pathways dysregulated early in the course of disease. This might offer mechanistic insights into disease pathogenesis and progression and have implications in therapeutic strategies. The six subjects had biopsy-proven diabetic nephropathy, lupus nephritis (*n* = 2), postacute tubular necrosis damage, NSAID nephropathy, and membranoproliferative glomerulonephritis, respectively. The mean estimated glomerular filtration rate (eGFR) was 83 mL/min in control subjects and 52 mL/min in CKD subjects.

### 3.2. Glycoprotein Spectral Count Normalization

Glycoproteins were extracted and enriched from the twelve urinary samples. To account for variations in the glycoprotein extraction efficiency, 5 *μ*g of the yeast protein invertase from *Saccharomyces cerevisiae* was added to each sample prior to extraction. After addition to the database, invertase spectral count served as a surrogate marker for extraction efficiency in each individual sample. Invertase spectral counts ranged from 31 to 122 in the twelve samples with an average spectral count of 86 (±31). Each sample was normalized independently to the invertase spectral counts.

To account for intersubject urine concentration variability, spectral counts were then normalized to urine creatinine content. This provides standardization for urinary creatinine excretion and concentration differences which can vary with volume status, stress, diet, activity level, age, gender, and overall health status [22]. Indeed, this normalization is commonly followed in clinical practice where degree of urinary protein is normalized to creatinine to obtain protein excretion rates [23]. Final spectral counts were expressed per mmol creatinine.

### 3.3. Urine Glycoproteome Is Altered in CKD

**Figure 1:**
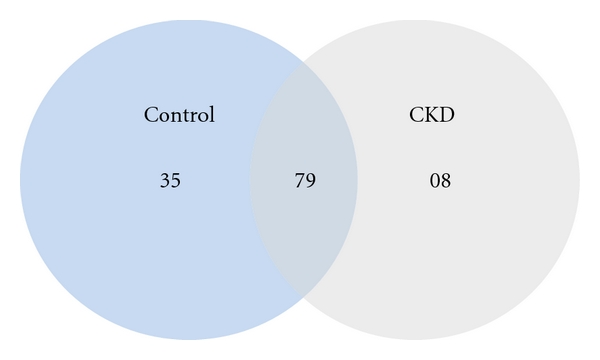
Venn diagram of the total urinary glycoproteins detected in healthy controls and CKD subjects. Tryptic digests of urine glycoproteins were subjected to LC-ESI-MS/MS analysis, and the proteins were identified as described in Section 2. 35 proteins were unique to healthy control subjects while 8 proteins were unique to subjects with CKD. 79 proteins were present in both groups.

Urinary glycoproteins were isolated from six subjects with CKD and six healthy controls using a hydrazide technique as described in Section 2. A total of 122 glycoproteins were identified, of which 35 proteins were unique to healthy control patients, 8 were unique to CKD subjects, and 79 were common proteins in both groups (Figure 1, Table 2). Unique proteins to the CKD group were Antithrombin-III (SERPINC1), Complement factor H-related 1 (CFHR1), Desmoglein-2 (DSG2), Lumican (LUM), Lymphatic vessel endothelial hyaluronic acid receptor 1 (LYVE1), Pigment epithelium-derived factor (SERPINF1), Thyroxine-binding globulin (SERPINA7), and Zinc-alpha-2-glycoprotein (AZP1). 

**Figure 2:**
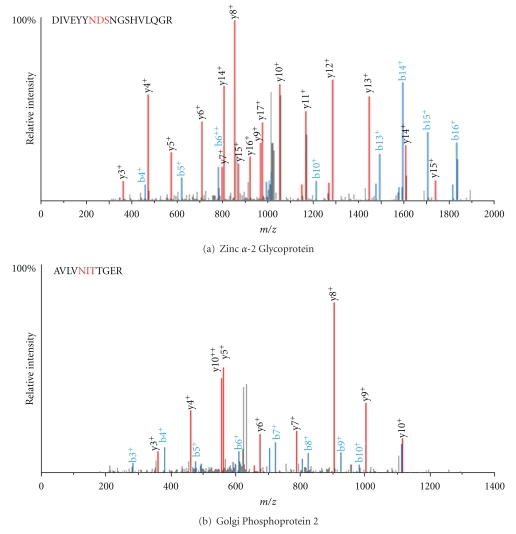
Mass Spectra of glycopeptides derived from Zinc alpha 2 Glycoprotein (a) and Golgi phosphoprotein (b) in CKD urine. Tryptic digests of urine glycoproteins were subjected to LC-ESI-MS/MS analysis as described in Section 2. The mass spectra of peptides DIVEYYNDSNGSHVLQG from zinc alpha 2 glycoprotein which is upregulated (a) and those of peptide AVLVNNITTGER from Golgi phosphoprotein which is significantly downregulated in CKD subjects (b) are shown. The N-linked glycosylation site of each peptide is depicted in red.

Figure 2 displays MS spectra of two individual glycopeptides with glycosylation motifs which were altered in CKD subjects. Zinc-alpha-2-glycoprotein is significantly upregulated in CKD (Figure 2(a)), while Golgi phosphoprotein is significantly downregulated in CKD (Figure 2(b)).  Table 3 displays motifs and specific peptide modifications for all unique 122 proteins. Proteins were only included if the peptides contained the NxS/T motif.

To test if proteins were significantly up- or downregulated in CKD, normalized spectral counts from the 6 CKD subjects were compared with those from the healthy controls. As sample size was small and spectral counts were not normally distributed, comparisons were made with the nonparametric Mann-Whitney test. As 122 proteins were being simultaneously tested, the FDR and corresponding *q*-values were determined to account for false positive results.  Table 4 displays 23 proteins which are differentially expressed in CKD utilizing an uncorrected *P* value threshold of less than 0.05. These proteins include 70 kDa lysosomal alpha-glucosidase (GAA), Apolipoprotein D (APOD), Alpha-2-HS-glycoprotein chain B (FETUA), Alpha-1-acid glycoprotein 1 (ORM1), Antithrombin-III (SERPINC1), Beta-galactosidase (GLB1), Ceruloplasmin (CP), Cubilin (CUBN), Epidermal growth factor (EGF), Epididymis secretory sperm binding protein Li 44a (E9KL23), Galectin-3-binding protein (LGALS3BP), Golgi phosphoprotein 2 (GOLPH2), Haptoglobin beta chain (HP), Ig gamma-1 chain C region (IGHG1), Ig gamma-2 chain C region (IGHG2), Kininogen 1 (KNG1), Leucine-rich alpha-2-glycoprotein (LRG), Plasma protease C1 inhibitor (SERPING1), Prostaglandin D2 synthase 21 kDa (PTGDS), Transferrin (TF), Trypstatin (AMBP), Uromodulin (UMOD), and Zinc-alpha-2-glycoprotein (AZGP1). Following correction for multiple comparisons, differential expression remained significant in 12 proteins (APOD, ORM1, FETUA, E9KL23, LGALS3BP, GOLPH2, HP, KNG1, LRG, SERPING1, PTGDS, AZGP1). Incidentally, not all unique proteins to CKD or healthy control groups had statistically significant up- or down-regulation. For example, lumican was not isolated in any healthy control subjects and was found in only three of the six CKD subjects. Thus, lumican is unique to CKD; however, as it was only seen in three CKD subjects, it was not significantly upregulated in CKD via nonparametric testing.

### 3.4. Gene Ontology Analysis Reveals Enrichment for Distinct Biological Functions of Differentially Expressed Urinary Glycoproteins

The 23 proteins with differential expression in CKD were subjected to a GO Database search and further analyzed with GO Tools [20, 21]. GO Term Finder (http://go.princeton.edu/cgi-bin/GOTermFinder) allowed for clustered identification of proteins annotated to specific GO biological process, location, and function classifications. A subsequent GO Term Mapper (http://go.princeton.edu/cgi-bin/GOTermMapper) analysis of significantly altered proteins was performed to bin the proteins to GO parent terms or GO Slim terms (http://www.geneontology.org/GO.slims.shtml). 

**Figure 3:**
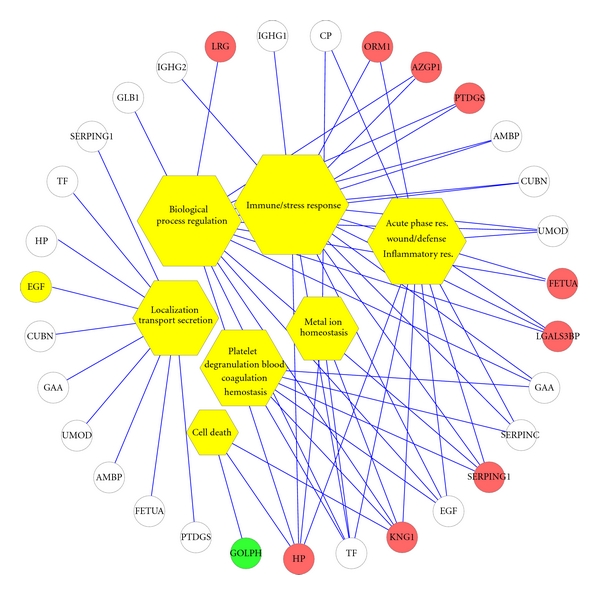
Global view of biological processes of differentially expressed urinary glycoproteins in CKD. Urinary glycoproteins that were differentially detected in CKD subjects were associated with biological functions using GO process annotations. This approach demonstrated significant overrepresentation of proteins involved in several categories, including regulation of response to stress, platelet activation/hemostasis/coagulation, acute-phase response, regulation of biological processes, localization, secretion, transport, and cell death. A bipartite network (generated using Cytoscape [24]) showing the relationship between GO process annotations (yellow hexagon nodes) and differentially regulated proteins in CKD subjects (white/red/green circular nodes). The size of the GO nodes is proportional to the number of edges (lines) that connect them to proteins. The 10 proteins that are altered with *q*-value of < 0.05 are depicted in red (up-regulation) and green (down-regulation) in CKD subjects. GAA, 70 kDa lysosomal alpha-glucosidase; APOD, Apolipoprotein D; FETUA: Alpha-2-HS-glycoprotein chain B; ORM1, Alpha-1-acid glycoprotein 1; SERPINC1, Antithrombin-III; GLB1, Beta-galactosidase; CP, Ceruloplasmin; CUBN, Cubilin; EGF, Epidermal growth factor; E9KL23, Epididymis secretory sperm binding protein Li 44a; LGALS3BP, Galectin-3-binding protein; GOLPH2, Golgi phosphoprotein 2; HP, Haptoglobin beta chain; IGHG1, Ig gamma-1 chain C region; IGHG2, Ig gamma-2 chain C region; KNG1, Kininogen 1; LRG, Leucine-rich alpha-2-glycoprotein; SERPING1,Plasma protease C1 inhibitor; PTGDS, Prostaglandin D2 synthase 21 kDa; TF, Transferrin; AMBP, Trypstatin; UMOD, Uromodulin; AZGP1, Zinc-alpha-2-glycoprotein.

GO analysis (Figure 3) for biological processes demonstrated that 16 of the 23 proteins were linked to immune/stress response and biological process regulation (*P* < 1 × 10^−4^). 9 of the 23 were acute-phase and inflammatory response proteins (*P* < 1 × 10^−3^). Six proteins were regulators of hemostasis, platelet degranulation and coagulation (*P* < 1 × 10^−4^), and 10 were involved in localization, transport, and secretion (*P* < 1 × 10^−4^). Other processes involved include metal ion homeostasis (4 proteins) and cell death (3 proteins). 

Table 5 displays function and location for the 23 proteins which were differentially expressed in CKD. 18 out of the 23 proteins localized to the extracellular region consistent with possible extracellular matrix remodeling that typifies renal disease. The analysis also revealed 2 major clusters of molecular function: 20 out of the 23 proteins were involved in binding and protein-protein interactions (*P* = 5 × 10^−4^). 5 proteins were endopeptidase inhibitors (*P* < 1 × 10^−6^). Collectively, these observations implicate the inflammatory/acute-phase response and extracellular matrix remodeling in CKD. They also strongly support the proposal that glycoproteomic analysis of urine might reveal mechanisms underpinning CKD.

## 4. Discussion

CKD is a growing public health problem with dramatic increases in morbidity and mortality following progression to ESRD. Given this, there is a tremendous need for the development of biomarkers to predict CKD progression and allow for early therapeutic intervention. Urine proteomic strategies are now at the forefront of this search due to the sensitivity of MS/MS analysis and the ability to develop noninvasive biomarkers from a readily available biofluid. Significant progress has been made, particularly in diabetes, where urine proteomic analysis can predict nephropathy [6, 25, 26]. Despite these developments, the majority of proteomic studies have relied on two-dimensional (2D) differential in-gel electrophoresis for protein separation. Resulting samples, particularly in CKD subjects, contain large amounts of highly abundant plasma proteins due to nonspecific leakage through the glomerular filtration barrier. Targeted analyses of low-abundance proteins will likely lead to more disease-specific and clinically relevant protein biomarkers. 

We therefore focused our attention on the urinary N-linked glycoproteome. Glycoproteins are an important protein subfraction accounting for up to 50% of the human proteome at any given time [27]. Due to their critical role in cell-cell interactions and signaling cascades, glycoproteins are promising markers for identifying kidney disease activity and progression. In this study we present an initial examination of the urinary N-linked glycoproteome in CKD subjects compared to healthy control subjects. We successfully isolated N-linked glycoproteins from twelve urine samples utilizing a hydrazide capture technique. 122 unique glycosylated proteins were detected amongst the twelve subjects (Table 3). This number is similar to other recent glycoproteome analyses. Ahn et al. recently reported isolating 164–174 unique proteins from human diabetic plasma using a multi-lectin column enrichment technique [16]. Yang et al. isolated 265 urinary glycoproteins from bladder cancer subjects and healthy controls also utilizing a multi-lectin column for enrichment, but larger sample sizes were used than in our current study [15]. These results support a successful hydrazide based technique for glycoprotein isolation in human urine. Further studies are required to identify optimal extraction strategies.

We detected 8 glycoproteins unique to CKD subjects and 35 unique to healthy controls (Table 2). Additionally, of the 122 total proteins identified, 23 glycoproteins were differentially expressed in CKD subjects versus healthy controls. 18 were upregulated in CKD while 5 were downregulated (Table 4). Many of the differentially expressed proteins have been previously linked to kidney disease supporting a potential role as a CKD biomarker. Two of the most significantly upregulated proteins in our CKD samples were AZGP1 and LRG, both of which are established inflammatory mediators. Alteration of AZGP1 and LRG expression is predictive of acute kidney injury in postsurgical patients [28]. AZGP1 has also been shown to be increased in diabetes and diabetic nephropathy [13, 29]. PTGDS, a known extracellular transporter for lipophilic molecules, is formed *de novo* in renal tubules [30]. PTGDS is upregulated in early diabetes [31] and is a marker of hypertension and latent renal injury [32]. SERPING1, an extracellular matrix regulator, is increased in acute renal allograft rejection perhaps suggesting an important role for collagen remodeling [33]. KNG1, a bradykinin precursor, has also been shown to be upregulated in acute renal allograft rejection [34], and gene variation induces altered aldosterone sensitivity in hypertensive subjects [35]. Interestingly, LUM, a proteoglycan, is a protein unique to CKD but without statistically significant up-regulation. Altered regulation of LUM has been linked with abnormal collagen fibril morphology as a mediator of fibrotic disease in diabetic nephropathy [36, 37]. CUBN, an apical protein in proximal tubule cells, was unique and downregulated in CKD. Recent investigation supports a role of CUBN in albumin reabsorption with genetic variance at this locus predicting microalbuminuria [38]. The decreased urinary CUBN excretion found in our CKD population may represent a dysfunctional variant or potentially a causative factor responsible for increasing proteinuria. 

We used annotations by the GO Consortium and GO Tools to connect the complex array of proteins identified in CKD subjects to biological processes, protein function, and cellular location. Many of the multiprotein pathways differentially expressed in CKD are involved in coagulation, inflammation, and acute-phase response (Table 5, Figure 3). Twenty proteins were linked to protein-protein interactions and binding. Remarkably, there were altered levels of proteins that were involved in acute-phase response and immune/stress response proteins (18 out of 23), implicating a possible mechanistic role for these pathways in CKD. Our detection of the several extracellular proteins and matrix remodeling proteases likely reflects matrix remodeling that occurs in CKD. These findings are consistent with previous literature, as CKD is known to have increased propensity for atherosclerosis, endothelial dysfunction, increased basal inflammation, and altered stress response [39, 40].

 In this study, we have established normalization techniques which will be essential to future urine glycoproteome analyses. To account for variations in the glycoprotein extraction efficiency of individual samples, yeast invertase (yeast glycoprotein with several glycopeptides) was added to each sample prior to extraction. In this way, glycopeptides derived from invertase serve as an internal marker for the extraction efficiency in each sample. Our samples were also normalized for urine creatinine content. This is of particular importance as marked intersubject variability can exist in creatinine excretion in random urine specimens consistent with different concentrations due to hydration status. Indeed, such normalization would be essential to extrapolate net excretion rates of a given protein in 24 hours and is commonly employed in clinical practice to quantify albumin excretion rates [23]. 

In summary, we have utilized a hydrazide-based approach to enrich the urinary glycoproteome with subsequent identification of the urinary glycoproteins in a human CKD population for the first time. Our results indicate that urine carries a distinct population of glycoproteins that function in proteinase inhibition, protein binding, and the acute-phase/immune-stress response in subjects with CKD. It will be of interest to study a larger number of subjects to determine whether urinary levels of these proteins might be useful indicators of CKD and to investigate the proposal that these proteins could be markers of disease progression.

## Figures and Tables

**Table 1:** Patient characteristics of study subjects.

Variable	Healthy control (*n* = 6)	CKD (*n* = 6)	*P*
Age (years)	46.3 (13.5)	47.2 (14.2)	0.92
Sex (male/female)	2/4	2/4	1.00
Body mass index (kg/m^2^)	24.3 (3.0)	30.5 (4.8)	0.02
Serum creatinine (mg/dL)	0.85 (0.16)	1.75 (1.09)	0.07
eGFR (mL/min)*	83.0 (15.0)	52.0 (27.4)	0.05
Protein/creatinine ratio	0.03 (0.02)	2.15 (1.44)	0.01

All data expressed as mean ± SD.

eGFR estimated glomerular filtration rate.

*eGFR calculated from Modification of Diet in Renal Disease formula.

**Table 2:** Urinary glycoproteins unique to CKD or healthy control subjects.

Unique proteins in healthy controls	Unique proteins in CKD
70 kDa lysosomal alpha-glucosidase (GAA)	Antithrombin-III (SERPINC1)
Alpha-1B-glycoprotein (A1BG)	Complement factor H-related 1 (CFHR1)
Basigin (BSG)	Desmoglein-2 (DSG2)
Beta-galactosidase (GLB1)	Lumican (LUM)
Beta-sarcoglycan (SGCB)	Lymphatic vessel endothelial hyaluronic acid receptor 1 (LYVE1)
Butyrophilin (BTN2A1)	Pigment epithelium-derived factor (SERPINF1)
Carboxypeptidase M (CPM)	Thyroxine-binding globulin (SERPINA7)
CD276 antigen (CD276)	Zinc-alpha-2-glycoprotein (AZP1)
Complement component C4B (C4B)	
Cubilin (CUBN)	
Colony stimulating factor 1 (macrophage) (CSF1)	
Delta and notch-like epidermal growth factor-related receptor (DNER)	
Desmocollin-2 (DSC2)	
Desmoglein-1 (DSG1)	
Epidermal growth factor (EGFR)	
Secreted frizzled-related protein-4 (SFRP4)	
Fibronectin 1 (FN1)	
Folate receptor alpha (FOLR1)	
Golgi phosphoprotein 2 (GOLPH2)	
Glutamyl aminopeptidase (ENPEP)	
Hepatitis B virus receptor binding protein (Q6PYX1)	
Hepatic asialoglycoprotein receptor 1 transcript variant b (ASGR1)	
Heparan sulfate proteoglycan 2 (HSPG2)	
Intercellular adhesion molecule 1 (ICAM1)	
Kallikrein-1 (KLK1)	
Kallikrein 3 (APS)	
Lysosomal alpha-glucosidase (GAA)	
Lysosomal-associated membrane protein 2 (LAMP2)	
Maltase-glucoamylase (MGAM)	
Microfibril-associated glycoprotein 4 (MFAP4)	
Mucin-6 (MUC6)	
Neuronal pentraxin receptor (NPTXR)	
Neuropilin and tolloid-like protein 1 (NETO1)	
Probable serine carboxypeptidase (CPVL)	
Sex hormone binding globulin (SHBG)	

**Table 3:** Glycoproteins identified with peptides carrying NxS/T motif.

No.	Protein	Charge state	Peptide sequence with NxS/T motif
1	155 kDa platelet multimerin (MMRN1)	2^+^ 2^+^	LQ**N[115]LT**LPT**N[115]AS**IK FNPGAESVVLS**N[115]S**TLK

2	70 kDa lysosomal alpha-glucosidase (GAA)	2^+^ 2^+^	GVFIT**N[115]ET**GQPLIGK LE**N[115]LS**SSEM[147****]GYTATLTR

3	Afamin (AFAM)	2^+^ 2^+^/3^+^	DIENF**N[115]ST**QK YAEDKF**N[115]ET**TEK

4	Aminopeptidase N (AMPN)	3^+^ 2^+^	KL**N[115]YT**LSQGHR **N[115]AT**LVNEADKLR

5	Attractin (ATRN)	2^+^	IDSTG**N[115]VT**NELR

6	Apolipoprotein D (APO D)	2^+^/3^+^ 2^+^	ADGTVNQIEGEATPV**N[115]LT**EPAK C[160****]IQA**N[115]YS**LM[147****]EN**[115]**GK

7	Apolipoprotein F (APO F)	2^+^	Q[111****]GGV**N[115]AT**QVLIQHLR

8	Apolipoprotein J (APO J)	2^+^ 2^+^/3^+^ 3^+^ 3^+^	LA**N[115]LT**QGEDQYYLR EDAL**N[115]ET**RESETK M[147****]L**N[115]TS**SLLEQLNEQFNWVSR EIRH**N[115]ST**GC160LR

9	Alpha-1-antichymotrypsin (AACT)	3^+^/4^+^ 3^+^/4^+^ 2^+^/3^+^ 2^+^/3^+^	GLKF**N[115]LT**ETSEAEIHQSFQHLLR YTG**N[115]AS**ALFILPDQDKM[147****]EEVEAM[147****]LLPETLKR TL**N[115]QS**SDELQLSM[147****]GNAM[147****]FVK KLIN[115****]DYVK**N[115]GT**R

10	Alpha-2-HS-glycoprotein (FETUA)	2^+^/3^+^ 2^+^/3^+^	AALAAFNAQN**N[115]GS**NFQLEEISR KVC[160****]QDC[160****]PLLAPL**N[115]DT**R

11	Alpha-1-acid glycoprotein 1 (ORM1)	2^+^/3^+^ 3^+^ 3^+^/4^+^	QDQC[160****]IY**N[115]TT**YLNVQR SVQEIQATFFYFTP**N[115]KT**EDTIFLR N[115****]EEY**N[115]KS**VQEIQATFFYFTP**N[115]KT**EDTIFLR

12	Alpha-1-acid glycoprotein 2 (ORM2)	2^+^/3^+^ 3^+^	QNQC[160****]FY**N[115]SS**YLNVQR SVQEIQATFFYFTP**N[115]KT**EDTIFLR

13	Alpha-1B-glycoprotein (A1BG)	3^+^/4^+^	EGDHEFLEVPEAQEDVEATFPVHQPG**N[115]YS**C[160****]SYR

14	Antithrombin-III (SERPINC1)	2^+^/3^+^ 2^+^	LGAC[160****]**N[115]DT**LQQLM[147****]EVFKFDTISEK SLTF**N[115]ET**YQDISELVYGAK

15	Basigin (BSG)	3^+^ 3^+^	ITDSEDKALM[147****]**N[115]GS**ESR ILLTC[160****]SL**N[115]DS**ATEVTGHR

16	Beta-galactosidase (GLB1)	2^+^	NNVITL**N[115]IT**GK

17	Beta-sarcoglycan (SGCB)	2^+^	ITS**N[115]AT**SDLNIK

18	Biotinidase (BTD)	2^+^/3^+^ 2^+^/3^+^	NPVGLIGAE**N[115]AT**GETDPSHSK DVQIIVFPEDGIHGF**N[115]FT**R

19	Butyrophilin, subfamily 2, member A1 (BTN2A1)	2^+^	GSVALVIH**N[115]IT**AQEN[115****]GTYR

20	Cathepsin D heavy chain (CTSD)	2^+^	GSLSYL**N[115]VT**R

21	Cathepsin L (CTSL)	3^+^	YSVA**N[115]DT**GFVDIPKQEK

22	Carboxypeptidase B2 (CBPB2)	2^+^/3^+^	QVHFFV**N[115]AS**DVDNVK

23	Carboxypeptidase M (CBPM)	2^+^ 4+	NFPDAFEYN**N[115]VS**R TVAQ**N[115]YS**SVTHLHSIGK

24	Calcium binding protein 39 (CAB39)	2^+^	H**N[115]FT**IM[147****]TK

25	CD276 antigen (CD276)	2^+^	VVLGA**N[115]GT**YSC[160****]LVR

26	CD163 antigen (CD163)	2^+^	APGWA**N[115]SS**AGSGR

27	CD44 protein (CD44)	2^+^	AF**N[115]ST**LPTM[147****]AQM[147****]EK

28	CD7 antigen (CD7)	3^+^	GRIDFSGSQD**N[115]LT**ITM[147****]HR

29	Cell adhesion molecule 1 (CADM1)	2^+^ 2^+^	VSLT**N[115]VS**ISDEGR FQLL**N[115]FS**SSELK

30	Ceruloplasmin (CP)	2^+^/3^+^ 3^+^/4^+^ 2^+^	EHEGAIYPD**N[115]TT**DFQR ELHHLQEQ**N[115]VS**NAFLDKGEFYIGSK E**N[115]LT**APGSDSAVFFEQGTTR

31	Complement component C4B (C4B)	2^+^	GL**N[115]VT**LSSTGR

32	Complement factor H (CFH)	3^+^	IPC[160****]SQPPQIEHGTI**N[115]SS**R

33	Complement factor H-related 1 (CFHR1)	2^+^	LQNNEN**N[115]IS**C[160****]VER

34	Complement factor I (CFI)	2^+^	FLN**N[115]GT**C[160****]TAEGK

35	Cubilin (CUBN)	2^+^ 2^+^	LC[160****]SSV**N[115]VS**NEIK AGF**N[115]AS**FHK

36	Corticosteroid-binding globulin (SERPINA6)	2^+^ 3^+^	AQLLQGLGF**N[115]LT**ER AVLQLNEEGVDTAGSTGVTL**N[115]LT**SKPIILR

37	Colony stimulating factor 1 (macrophage) (CSF1)	2^+^	VKNVF**N[115]ET**K

38	Delta and notch-like epidermal growth factor-related receptor (DNER)	2^+^	LVSFEVPQ**N[115]TS**VK

39	Desmocollin-2 (DSC2)	2^+^ 2^+^	LKAI**N[115]DT**AAR A**N[115]YT**ILK

40	Desmoglein-1 (DSC1)	2^+^	DYNTK**N[115]GT**IK

41	Desmoglein-2 (DSG2)	2^+^ 2^+^	I**N[115]AT**DADEPNTLNSK YVQ**N[115]GT**YTVK

42	DNA ligase 4 (LIG4)	2^+^	AP**N[115]LT**NVNK

43	Dual specificity protein phosphatase CDC14B (CDC14B)	2^+^	NH**N[115]VT**TIIR

44	Epidermal growth factor (EGF)	**2^+^**	G**N[115]NS**HILLSALK

45	Epididymis secretory sperm binding protein Li 44a (SERPINA1)	2^+^/3^+^/4^+^ 3^+^/4^+^	YLG**N[115]AT**AIFFLPDEGKLQHLENELTHDIITK ADTHDEILEGLNF**N[115]LT**EIPEAQIHEGFQELLR

46	Extracellular link domain containing 1 (XLKD1)	2^+^/3^+^	KANQQL**N[115]FT**EAK

47	Fibrillin 1 (FBN1)	**2^+^**	TAIFAF**N[115]IS**HVSNK

48	Fibrinopeptide A (FGA)	**2^+^**	M[147****]DGSLNF**N[115]RT**

49	Fibronectin type III domain-containing protein 5 (FNDC5)	**2^+^**	FIQEV**N[115]TT**TR

50	Frizzled protein 4 (FRP4)	**2^+^**	ISM[147****]C[160****]QNLGY**N[115]VT**K

51	Fibronectin 1 (FN1)	3^+^	DQC[160****]IVDDITYNV**N[115]DT**FHK

52	Folate receptor alpha (FOLR1)	**2^+^**	GW**N[115]WT**SGFNK

53	Galectin-3-binding protein (LGALS3BP)	2^+^ 2^+^ 2^+^ 2^+^	ALGFE**N[115]AT**QALGR AAIPSALDT**N[115]SS**K GL**N[115]LT**EDTYKPR TVIRPFYLT**N[115]SS**GVD

54	Glutaminyl-peptide cyclotransferase (QPCT)	2^+^/3^+^ 3^+^/4^+^	NYHQPAIL**N[115]SS**ALR YFQ**N[115]YS**YGGVIQDDHIPFLR

55	Golgi phosphoprotein 2 (GOLPH2)	3^+^ 1^+^/2^+^	LQQDVLQFQKN[115****]QTNLER AVLVN[115****]**N[115]IT**TGER

56	Glutamyl aminopeptidase (ENPEP)	**2^+^**	HTAEYAA**N[115]IT**K

57	Haptoglobin beta chain (HP)	2^+^/3^+^ 3^+^/4^+^ 2^+^/3^+^/4^+^ 3^+^	VVLHP**N[115]YS**QVDIGLIK MVSHH**N[115]LT**TGATLINEQWLLTTAK NLFL**N[115]HS**E**N[115]AT**AKDIAPTLTLYVGKK Q[111****]LVEIEKVVLHP**N[115]YS**QVDIGLIK

58	HEG homolog 1 (HEG1)	**2^+^**	SYSESSSTSSSESL**N[115]SS**APR

59	Hemopexin (HPX)	3^+^/4^+^ 2^+^	GHGHR**N[115]GT**GHG**N[115]ST**HHGPEYM[147****]R ALPQPQ**N[115]VT**SLLGC[160****]TH

60	Hepatitis B virus receptor binding protein (Q6YPX1)	2^+^	EEQY**N[115]ST**YR

61	Hepatic asialoglycoprotein receptor 1 transcript variant b (ASGR1)	2^+^	ETFS**N[115]FT**ASTEAQVK

62	Heparan sulfate proteoglycan 2 (HSPG2)	2^+^	ALV**N[115]FT**R

63	Ig alpha-1 chain C region (IGHA1)	3^+^	LAGKPTHV**N[115]VS**VVM[147****]AEVDGTC[160****]Y

64	Ig gamma-1 chain C region (IGHG1)	2^+^ 2^+^/3^+^	EEQY**N[115]ST**YR TKPREEQY**N[115]ST**YR

65	Ig gamma-2 chain C region (IGHG2)	2^+^ 2^+^/3^+^	EEQF**N[115]ST**FR TKPREEQF**N[115]ST**FR

66	Ig gamma-4 chain C region (IGHG4)	2^+^ 2^+^/3^+^	EEQF**N[115]ST**FR TKPREEQF**N[115]ST**FR

67	Ig mu chain C region (IGHM)	2^+^	YK**N[115]NS**DISSTR

68	Inducible T-cell co-stimulator ligand (ICOSLG)	2^+^	TVVTYHIPQ**N[115]SS**LENVDSR

69	Insulin-like growth factor-binding protein 3 (IGFBP3)	2^+^	GLC[160****]V**N[115]AS**AVSR

70	Intercellular adhesion molecule 1 (ICAM1)	2 2^+^	LNPTVTYG**N[115]DS**FSAK A**N[115]LT**VVLLR

71	Intercellular adhesion molecule 2 (ICAM2)	2^+^	G**N[115]ET**LHYETFGK

72	Kallikrein-1 (KLK1)	4^+^	HNLFDDEN[115****]TAQFVHVSESFPHPGF**N[115]M[147]S**LLE**N[115]HT**R

73	KALLIKREIN-2 (KLK2)	2^+^	**N[115]KS**VILLGR

74	Kininogen 1 (KNG1)	2^+^ 2^+^ 3^+^/4^+^ 2^+^	LNAEN**N[115]AT**FYFK ITYSIVQT**N[115]C[160]S**K HGIQYFN**N[115]NT**QHSSLFTLNEVKR YNSQ**N[115]QS**NNQFVLYR

75	Leucine-rich alpha-2-glycoprotein (LRG1)	2^+^ 2^+^/3^+^	MFSQ**N[115]DT**R KLPPGLLA**N[115]FT**LLR

76	Leukocyte-associated immunoglobulin-like receptor 1 (LAIR1)	2^+^/3^+^	STY**N[115]DT**EDVSQASPSESEAR

77	Lumican (LUM)	3^+^	KLHINHN**N[115]LT**ESVGPLPK

78	Lysosomal acid phosphatase (ACP2)	2^+^	YEQLQ**N[115]ET**R

79	Lysosomal alpha-glucosidase (GAA)	2^+^ 2^+^	GVFIT**N[115]ET**GQPLIGK LE**N[115]LS**SSEM[147****]GYTATLTR

80	Lysosome-associated membrane glycoprotein 1 (LAMP1)	2^+^	GHTLTL**N[115]FT**R

81	Lysosomal-associated membrane protein 2, (LAMP2)	2^+^	VASVININP**N[115]TT**HSTGSC[160****]R

82	Lymphatic vessel endothelial hyaluronic acid receptor 1 (XLKD1)	2^+^	ANQQL**N[115]FT**EAK

83	Lysyl oxidase (LOX)	3^+^ 3^+^	AE**N[115]QT**APGEVPALSNLRPPSR RDPGAAVPGAA**N[115]AS**AQQPR

84	Major prion protein (PRNP)	2^+^	Q[111****]HTVTTTTKGE**N[115]FT**ETDVK

85	Membrane protein FAM174A (FAM174A)	2^+^	GSEGG**N[115]GS**NPVAGLETDDHGGK

86	Maltase-glucoamylase (MGAM)	2^+^ 2^+^	ILGM[147****]EEPS**N[115]VT**VK VILILDPAISG**N[115]ET**QPYPAFTR

87	Microfibril-associated glycoprotein 4 (MFAP4)	2^+^	VDLEDFE**N[115]NT**AYAK

88	Monocyte differentiation antigen CD14	2^+^	LR**N[115]VS**WATGR

89	Mucin-6 (MUC6)	2^+^	GC[160****]M[147****]A**N[115]VT**VTR

90	N-acetylglucosamine-6- sulfatase (GNS)	2^+^ 2^+^	YY**N[115]YT**LSIN[115****]GK TPMT**N[115]SS**IQFLDNAFR

91	N-acylsphingosine amidohydrolase (ASAH1)	2^+^	TVLE**N[115]ST**SYEEAK

92	Neuronal pentraxin receptor (NPTXR)	2^+^	ALPGGAD**N[115]AS**VASGAAASPGPQR

93	Neuropilin and tolloid-like protein 1 (NETO1)	2^+^	HESEY**N[115]TT**R

94	Peptidase inhibitor 16 (PI16)	2^+^	SLPNFP**N[115]TS**ATA**N[115]AT**GGR

95	Pigment epithelium-derived factor (SERPINF1)	3^+^	VTQ**N[115]LT**LIEESLTSEFIHDIDR

96	Plasma protease C1 inhibitor (SERPING1)	2^+^/3^+^ 2^+^ 3^+^	GVTSVSQIFHSPDLAIRDTFV**N[115]AS**R VLS**N[115]NS**DANLELINTWVAK VGQLQLSH**N[115]LS**LVILVPQNLK

97	Plasma serine protease inhibitor (SERPINA5)	2^+^	VVGVPYQG**N[115]AT**ALFILPSEGK

98	Platelet-derived growth factor subunit B (PDGFB)	3^+^	LLHGDPGEEDGAELDL**N[115]M[147]T**R

99	Polytrophin (TROPH)	2^+^	N[115****]**N[115]VT**EDIK

100	Probable G-protein coupled receptor 116 (GPR116)	2^+^ 2^+^	ANEQVVQSL**N[115]QT**YK YEEQQLEIQ**N[115]SS**R

101	Probable serine carboxypeptidase (CPVL)	2^+^	Q[111****]AIHVG**N[115]QT**FNDGTIVEK

102	Prosaposin (PSAP)	2^+^/3^+^	NLEK**N[115]ST**KQEILAALEK

103	Prostaglandin D2 synthase 21 kDa (PTGDS)	2^+^/3^+^ 2^+^	SVVAPATDGGL**N[115]LT**STFLR WFSAGLAS**N[115]SS**WLR

104	Prostatic acid phosphatase (ACPP)	3^+^	FL**N[115]ES**YKHEQVYIR

105	Proteinase-activated receptor 1 (F2R)	2^+^	AT**N[115]AT**LDPR

106	Protein shisa-7 (SHISA7)	2^+^	LTGALTGGGGAASPGA**N[115]GT**R

107	RING finger protein 10 (RNF10)	2^+^	**N[115]ES**FN[115****]**N[115]QS**R

108	Secretory component (Polymeric IG Receptor) (PIGR)	3^+^ 2^+^ 2^+^ 2^+^	A**N[115]LT**NFPE**N[115]GT**FVVNIAQLSQDDSGR Q[111****]IGLYPVLVIDSSGYVNP**N[115]YT**GR VPG**N[115]VT**AVLGETLK YKCGLGI**N[115]S**R

109	Slit homolog 1 (SLIT1)	2^+^	LELN[115****]GN[115****]**N[115]IT**R

110	Sushi domain-containing protein 2 (SUSD2)	2^+^	SELV**N[115]ET**R

111	Sex hormone binding globulin (SHBG)	2^+^	LDVDQAL**N[115]RT**

112	Transferrin (TF)	2^+^/3^+^ 2^+^/3^+^	Q[111****]QQHLFGS**N[115]VT**DC[160****]SGNFC[160****]LFR C[160****]GLVPVLAENY**N[115]KS**DN[115****]C[160****]EDTPEAGYFAVAVVK

113	Thrombin heavy chain (F2)	4^+^	YPHKPEI**N[115]ST**THPGADLQENFC[160****]R

114	Tripeptidyl-peptidase I variant (TPP1)	3^+^	FLSSSPHLPPSSYFN[115****]ASGR

115	Tyrosine-protein kinase receptor UFO (AXL)	2^+^ 3^+^	SLHVPGL**N[115]KT** **N[115]GS**QAFVHWQEPR

116	TIMP metallopeptidase inhibitor 1 (TIMP1)	2^+^ 3^+^	FVGTPEV**N[115]QT**TLYQR SH**N[115]RS**EEFLIAGK

117	Thyroxine-binding globulin (SERPINA7)	2^+^	TLYETEVFSTDFS**N[115]IS**AAK

118	Trypstatin (AMBP)	2^+^/3^+^	SKW**N[115]IT**M[147****]ESYVVHTNYDEYAIFLTK

119	Transmembrane protein 108 (TMEM108)	4^+^	KGAG**N[115]SS**RPVPPAPGGHSR

120	Uromodulin (UMOD)	2^+^/3^+^ 2^+^ 2^+^/3^+^	Q[111****]DF**N[115]IT**DISLLEHR FALLMTNCYATPSS**N[115]AT**DPLK CNTAAPMWL**N[115]GT**HPSSDEGIVSR

121	Vasorin (VASN)	2^+^	LHEIT**N[115]ET**FR

122	Zinc-alpha-2-glycoprotein (AZGP1)	2^+^/3^+^ 3^+^/4^+^ 2^+^	DIVEYY**N[115]DSN[115]GS**HVLQGR AREDIFM[147****]ETLKDIVEYY**N[115]DSN[115]GS**HVLQGR FGCEIEN**N[115]RS**

**Table 4:** Differentially regulated proteins identified in CKD subjects.

Protein Code	Name of the protein identified	*P* value	*q*-value	Direction of change in CKD subjects
APOD	Apolipoprotein D	0.0022	0.0224	Up
FETUA	Alpha-2-HS-glycoprotein chain B	0.0022	0.0224	Up
ORM1	Alpha-1-acid glycoprotein 1	0.0022	0.0224	Up
E9KL23	Epididymis secretory sperm binding protein Li 44a	0.0022	0.0224	Up
LGALS3BP	Galectin-3-binding protein	0.0022	0.022	Up
GOLPH2	Golgi phosphoprotein 2	0.0022	0.0224	Down
HP	Haptoglobin beta chain	0.0022	0.0224	Up
KNG1	Kininogen 1	0.0022	0.0224	Up
LRG	Leucine-rich alpha-2-glycoprotein	0.0022	0.0224	Up
SERPING1	Plasma protease C1 inhibitor	0.0022	0.0224	Up
PTGDS	Prostaglandin D2 synthase 21kDa	0.0022	0.0224	Up
AZGP1	Zinc-alpha-2-glycoprotein	0.0022	0.0224	Up
GAA	70 kDa lysosomal alpha-glucosidase	0.0152	0.13	Down
SERPINC1	Antithrombin-III	0.0152	0.103	Up
GLB1	Beta-galactosidase	0.0152	0.103	Down
CUBN	Cubilin	0.0152	0.103	Down
EGF	Epidermal growth factor	0.0152	0.13	Down
UMOD	Uromodulin	0.0152	0.103	Up
TF	Transferrin	0.0216	0.1387	Up
AMBP	Trypstatin	0.0411	0.18	Up
CP	Ceruloplasmin	0.0433	0.18	Up
IGHG1	Ig gamma-1 chain C region	0.0433	0.18	Up
IGHG2	Ig gamma-2 chain C region	0.0433	0.18	Up

**Table 5:** Component location and function associated with significantly up- or downregulated proteins identified in CKD patients.

Location					Function				
Gene ontology term	Cluster frequency	*P*-value	FDR	Proteins annotated to the GO Term	Gene ontology term	Cluster frequency	*P* value	FDR	Proteins annotated to the GO term
Extracellular region	18 of 23 proteins, 78.3%	7.36*E*−15	0.00%	EGF, IGHG1, LRG, AMBP, AZGP1, KNG1, SERPING1, TF, LGALS3BP, FETUA, CP, HP, SERPINC1, IGHG2, ORM1, APOD, PTGDS, UMOD	Binding	20 of 23 proteins, 87.0%	0.00051	0.89%	EGF, IGHG1, AMBP, CUBN, AZGP1, KNG1, SERPING1, TF, LGALS3BP, GAA, FETUA, CP, SERPINC1, HP, IGHG2, GLB1, ORM1, APOD, PTGDS, UMOD
Extracellular space	11 of 23 proteins, 47.8%	5.47*E*−11	0.00%	EGF, LGALS3BP, CP, FETUA, SERPINC1, APOD, ORM1, PTGDS, UMOD, KNG1, SERPING1	Protein binding	15 of 23 proteins, 65.2%	0.00066	0.80%	TF, EGF, IGHG1, LGALS3BP, CP, FETUA, AMBP, HP, SERPINC1, APOD, ORM1, GLB1, CUBN, KNG1, SERPING1
Extracellular region part	11 of 23 proteins, 47.8%	1.13*E*−09	0.00%	EGF, LGALS3BP, CP, FETUA, SERPINC1, APOD, ORM1, PTGDS, UMOD, KNG1, SERPING1	Enzyme regulator activity	6 of 23 proteins, 26.1%	0.00024	0.00%	EGF, SERPINC1, FETUA, KNG1, AMBP, SERPING1
Cytoplasmic vesicle	6 of 23 proteins, 26.1%	6.81*E*−05	0.20%	TF, EGF, CUBN, UMOD, KNG1, SERPING1	Endopeptidase inhibitor activity	5 of 23 proteins, 21.7%	3.78*E*−07	0.00%	SERPINC1, FETUA, KNG1, AMBP, SERPING1
Vesicle	6 of 23 proteins, 26.1%	8.57*E*−05	0.18%	TF, EGF, CUBN, UMOD, KNG1, SERPING1	Endopeptidase regulator activity	5 of 23 proteins, 21.7%	4.29*E*−07	0.00%	SERPINC1, FETUA, KNG1, AMBP, SERPING1
Cell fraction	5 of 23 proteins, 21.7%	0.00405	2.29%	EGF, IGHG2, IGHG1, CUBN, AMBP	Peptidase inhibitor activity	5 of 23 proteins, 21.7%	4.84*E*−07	0.00%	SERPINC1, FETUA, KNG1, AMBP, SERPING1
Cytoplasmic membrane-bounded vesicle	5 of 23 proteins, 21.7%	0.00055	0.15%	TF, EGF, CUBN, KNG1, SERPING1	Peptidase regulator activity	5 of 23 proteins, 21.7%	1.10*E*−06	0.00%	SERPINC1, FETUA, KNG1, AMBP, SERPING1
Membrane-bounded vesicle	5 of 23 proteins, 21.7%	0.00062	0.29%	TF, EGF, CUBN, KNG1, SERPING1	Enzyme inhibitor activity	5 of 23 proteins, 21.7%	8.50*E*−06	0.00%	SERPINC1, FETUA, KNG1, AMBP, SERPING1
Cytoplasmic vesicle part	4 of 23 proteins, 17.4%	0.00024	0.17%	EGF, CUBN, KNG1, SERPING1	Transporter activity	5 of 23 proteins, 21.7%	0.00527	2.88%	CUBN, APOD, AZGP1, PTGDS, AMBP
Stored secretory granule	4 of 23 proteins, 17.4%	5.33*E*−05	0.22%	TF, EGF, KNG1, SERPING1	Carbohydrate binding	3 of 23 proteins, 13.0%	0.00656	2.50%	SERPINC1, GAA, KNG1
Membrane fraction	4 of 23 proteins, 17.4%	0.00796	2.69%	IGHG2, IGHG1, CUBN, AMBP	Lipid binding	3 of 23 proteins, 13.0%	0.0092	2.82%	APOD, AZGP1, PTGDS
Insoluble fraction	4 of 23 proteins, 17.4%	0.00897	2.81%	IGHG2, IGHG1, CUBN, AMBP	Serine-type endopeptidase inhibitor activity	3 of 23 proteins, 13.0%	9.99*E*−05	0.00%	SERPINC1, AMBP, SERPING1, E9KL23
Apical plasma membrane	3 of 23 proteins, 13.0%	0.00069	0.27%	TF, CUBN, UMOD	Hemoglobin binding	2 of 23 proteins, 8.7%	1.36*E*−05	0.00%	HP, CUBN
Perinuclear region of cytoplasm	3 of 23 proteins, 13.0%	0.00565	2.58%	TF, GLB1, PTGDS	Fatty acid binding	2 of 23 proteins, 8.7%	0.00076	0.73%	AZGP1, PTGDS
Apical part of cell	3 of 23 proteins, 13.0%	0.00146	0.94%	TF, CUBN, UMOD	Cysteine-type endopeptidase inhibitor activity	2 of 23 proteins, 8.7%	0.00084	0.83%	FETUA, KNG1
Lytic vacuole	3 of 23 proteins, 13.0%	0.0026	1.89%	CUBN, GLB1, GAA	Monocarbox-ylic acid binding	2 of 23 proteins, 8.7%	0.00161	0.92%	AZGP1, PTGDS
Lysosome	3 of 23 proteins, 13.0%	0.0026	1.79%	CUBN, GLB1, GAA	Hydrolase activity, hydrolyzing O-glycosyl compounds	2 of 23 proteins, 8.7%	0.00354	2.29%	GLB1, GAA
Platelet alpha granule lumen	3 of 23 proteins, 13.0%	2.55*E*−05	0.00%	EGF, KNG1, SERPING1	Protein kinase regulator activity	2 of 23 proteins, 8.7%	0.00418	2.13%	EGF, FETUA
Secretory granule lumen	3 of 23 proteins, 13.0%	1.37*E*−05	0.00%	EGF, KNG1, SERPING1	Kinase regulator activity	2 of 23 proteins, 8.7%	0.00541	2.71%	EGF, FETUA
Cytoplasmic membrane-bound vesicle lumen	3 of 23 proteins, 13.0%	1.55*E*−05	0.00%	EGF, KNG1, SERPING1	Hydrolase activity, acting on glycosyl bonds	2 of 23 proteins, 8.7%	0.0056	2.78%	GLB1, GAA
Vesicle lumen	3 of 23 proteins, 13.0%	1.74*E*−05	0.00%	EGF, KNG1, SERPING1	Tetrapyrrole binding	2 of 23 proteins, 8.7%	0.00841	2.67%	CUBN, AMBP
Platelet alpha granule	3 of 23 proteins, 13.0%	2.55*E*−05	0.00%	EGF, KNG1, SERPING1	Heparin binding	2 of 23 proteins, 8.7%	0.00608	2.63%	SERPINC1, KNG1
Vacuole	3 of 23 proteins, 13.0%	0.00423	2.27%	CUBN, GLB1, GAA					
Extrinsic to membrane	2 of 23 proteins, 8.7%	0.00532	2.61%	CUBN, UMOD					
Lysosomal membrane	2 of 23 proteins, 8.7%	0.00699	2.64%	CUBN, GAA					
Endocytic vesicle	2 of 23 proteins, 8.7%	0.00332	2.10%	TF, CUBN					
Coated pit	2 of 23 proteins, 8.7%	0.00113	0.88%	TF, CUBN					
Cell projection membrane	2 of 23 proteins, 8.7%	0.00899	2.71%	CUBN, UMOD					

## Abbreviations

CKD: Chronic kidney disease
ESRD: End-stage renal disease
FDR: False discovery rate
GO: Gene ontology
LC-ESI-MS/MS: Liquid chromatography-electrospray ionization tandem MS analysis
MS: Mass spectrometry.
